# Early harvesting improves seed vigour of hybrid rice seeds

**DOI:** 10.1038/s41598-018-29021-5

**Published:** 2018-07-23

**Authors:** Xiaomin Wang, Huabin Zheng, Qiyuan Tang

**Affiliations:** College of agronomy, Hunan Agricultural University, Changsha, 410128 China

## Abstract

Maturity stage in harvesting time greatly affects seed vigour. This work aimed to scientific harvesting time of hybrid rice for being high vigour with high & stable seed yield. Field experiments of different harvesting time were conducted in 2013–2014, and germination percentage (GP), vigour index (VI), seed moisture content and 1000-grain weight was determined. Both GP and VI progressively increased to peaks and then began to decline with harvesting time delayed, and the regression coefficients of varieties were ranged from 0.7214 to 0.9066. In addition, difference values between tangent points (ΔX) of GP were higher than that of VI according to the quadratic functions. Based on seed vigour through the divided range from 75% to 125% of peak value, optimum harvesting time of IIY-416, JY-167, Yliangyou-1 (YLY-1) ranged from 17 to 27, 15 to 23 and 17 to 23 days after the completion of artificial pollination (DACAP), respectively. Moreover, when seedlots harvested from 17 to 23 DACAP, no significant difference was found on 1000-grain weight and the seed moisture content was kept relatively low (19–25%). Therefore, it can be concluded that hybrid rice seed can be earlier-harvest based on seed vigour, and 17 to 23 DACAP can be recommended as optimum harvesting time during hybrid rice seed production.

## Introduction

Rice (*Oryza sativa* L.) is the staple food for a large segment of the world population^1^. The hybrid rice seed production technology is one of the most successful application that increased rice yield potential by 15–20% and guaranteed greatly the Chinese food security in the past 30 years^2,3^. However, the price of hybrid rice seeds was higher (6.5$/kg–16.1$/kg) without after-sale service and technical support in China^4^. In additional, labor market and hybrid rice seed quality are greatly unstable. Consequently, it is urgent to improve seed quality of hybrid rice in order to satisfy low-input and mechanized cultivation during the transition period in China^4^. It is necessary to investigate and evaluate the quality of hybrid rice seeds.

Seed vigour is one of the key components of seed quality that provides accurate information on the field performance potential of seedlots^5,6^. High vigour seeds can resist the negative impact of variable environmental conditions during production and processing^5^. Furthermore, seeds with high vigour for companies ensure the competitive position in agricultural markets^5^. Generally, harvesting time is one of the important factors for affecting seed vigour^7,8^. Early harvesting seeds are immature and poorly developed, resulting in poor quality that affects subsequent storability compared to seeds harvested at physiological maturity. Delayed harvesting also leads to the loss of yield due to shattering, damage of seed and the risk of rain that affect seed quality^9^. Therefore, scientific period of harvesting time was a key of high seed vigour with stable & high seed yield. The influences of harvesting time on the characteristics of seed maturation have been reported by several researches. For example, Shaheb *et al*.^9^ reported that seed quality parameters, such as seed germination percentage (GP) and vigour index (VI), were significantly influenced by harvesting time. Kumar *et al*.^10^ found that seed yield and quality largely depended on the stage of maturity of crops. However, hybrid rice seeds are harvested based on personal experience for seed companies and farmers in China, which results in poor seed quality and grain yield due to improper harvesting time^11^. In present study, seed vigour parameters, including GP, VI, seed moisture content and 1000-grain weight were determined with optimizing harvesting time for being high seed vigour.

## Results

### Variation on germination percentage and vigour index with harvesting time delayed

**Figure 1:**
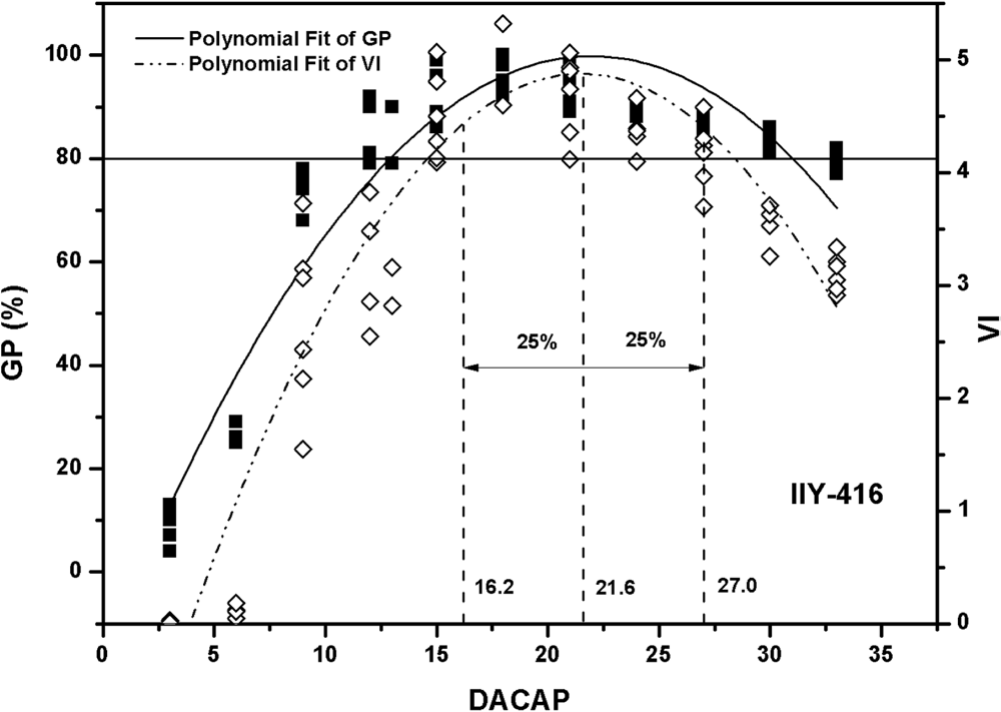
Polynomial fit of germination percentage (GP) and vigour index (VI) of IIY-416. IIY-416 indicates IIyou416. DACAP indicates days after the completion of artificial pollination. Three vertical dashed lines indicate abscissas of 75% of peak-value, peak value, 125% of peak-value, respectively. The horizontal solid line indicates 80% germination percentage, which is Chinese standard.

**Figure 2:**
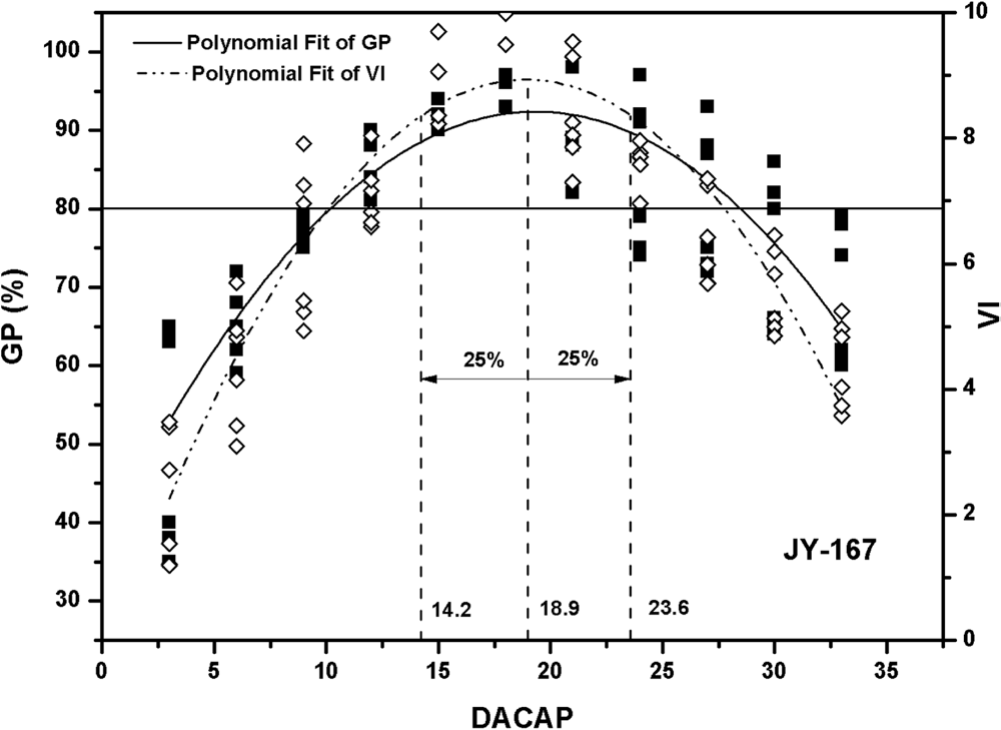
Polynomial fit of germination percentage (GP) and vigour index (VI) of JY-167. JY-167 indicates Jinyou167. DACAP indicates days after the completion of artificial pollination. Three vertical dashed lines indicate abscissas of 75% of peak-value, peak value, 125% of peak-value, respectively. The horizontal solid line indicates 80% germination percentage, which is Chinese standard.

**Figure 3:**
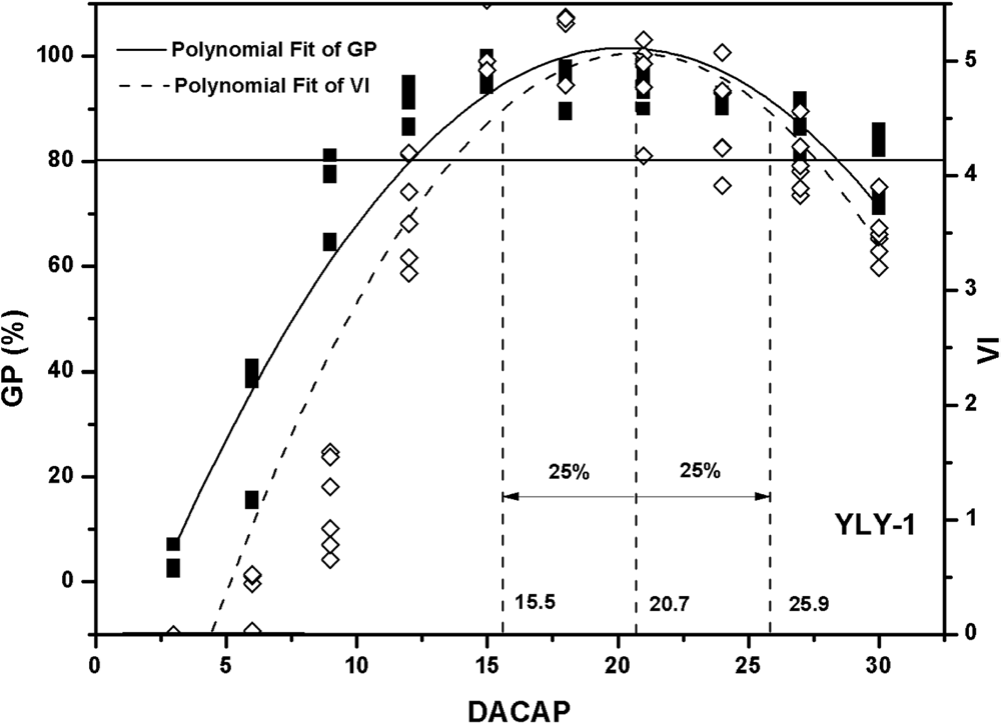
Polynomial fit of germination percentage (GP) and vigour index (VI) of YLY-1. YLY-1 indicates Yliangyou-1. DACAP indicates days after the completion of artificial pollination. Three vertical dashed lines indicate abscissas of 75% of peak-value, peak value, 125% of peak-value, respectively. The horizontal solid line indicates 80% germination percentage, which is Chinese standard.

A significant quadratic function was observed between germination percentage (GP), vigour index (VI) and days after the completion of artificial pollination (DACAP). Both GP and VI progressively increased to peaks and then began to decline as harvesting time delayed (Figs 1–3). The regression coefficients of GP among varieties were varied from 0.7214 to 0.9066 and averaged 0.8443. The regression coefficients of VI of varieties ranged from 0.8167 to 0.8616, showing a mean of 0.8433. In addition, difference values between tangent points (ΔX) of GP were higher than that of VI (Table 2). For example, in JY-167, two tangent points (X1 and X2) of GP were 9.9395 and 52.0223, respectively, but that of VI were 1.4735 and 38.2765, respectively. Moreover, through the analysis of parameter ΔX, it showed that seed vigour is more sensitive to harvesting time than GP.

In present experiment, the optimal harvesting time was determined based on seed vigour varied from 75% to 125% of peak value (Figs 1–3). Optimum harvesting time of IIY-416, JY-167, YLY-1 ranged from 17 to 27, 15 to 23 and 17 to 23 DACAP, respectively. Averaging across three varieties, the optimum harvesting time was ranged from 17 to 23 DACAP.

### Variation on 1000-grain weight and seed moisture content with harvesting time delayed

**Figure 4:**
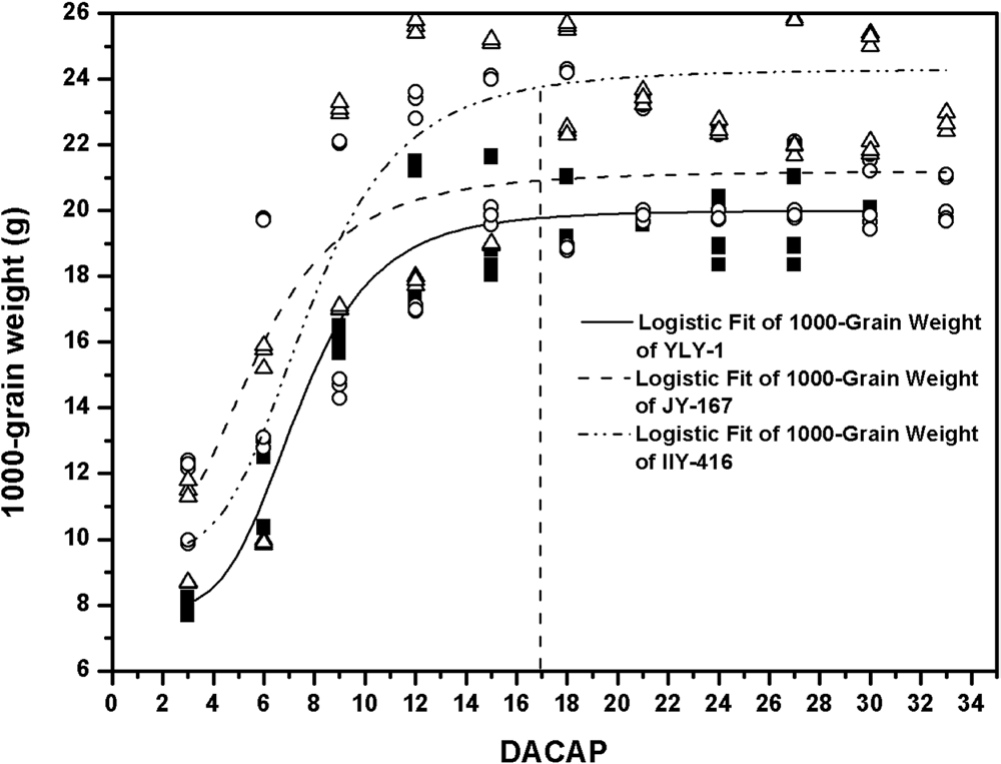
Logistic fit of 1000-grain weight of varieties. YLY-1, JY-167 and IIY-416 indicate Yliangyou-1, Jinyou167 and IIyou416, respectively. DACAP indicates days after the completion of artificial pollination.

A significant logistic relationship was obtained between 1000-grain weight and harvesting time (Fig. 4). The regression coefficients of varieties varied from 0.6101 to 0.9294, with an average of 0.7691 (Table 3). In addition, significant variations were observed on grain weight among different varieties, and maximum 1000-grain weight was observed in IIY-416. Moreover, no difference was found on 1000-grain weight among the seeds harvested after 17 days after the completion of artificial pollination (DACAP).

**Figure 5:**
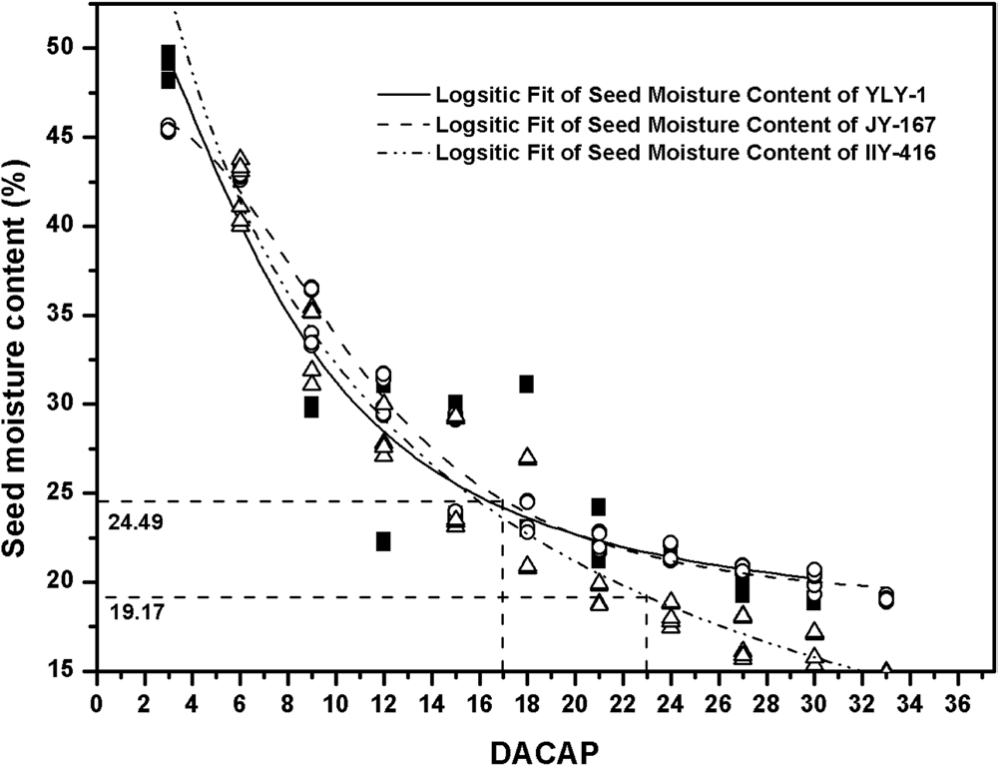
Logistic fit of seed moisture content of varieties. YLY-1, JY-167 and IIY-416 indicate Yliangyou-1, Jinyou167 and IIyou416, respectively. DACAP indicates days after the completion of artificial pollination.

A significant logistic relationship was observed between seed moisture content and harvesting time (Fig. 5). The regression coefficients among varieties ranged from 0.9020 to 0.9823 and averaged 0.9536 (Table 4). when seeds of the tested varieties harvested from 17 to 23 DACAP, the variation range of seed moisture content was 19%-25% (Fig. 5). The minimum seed moisture content (19.17%) was obtained when IIY-416 was harvested at 23 DACAP. The maximum seed moisture content (24.49%) was observed when JY-167 was harvested at 17 DACAP.

## Discussion

Seed quality can be measured by several aspects, such as viability, seedlot purity, health, and mechanical damage. Moreover, seed vigour plays essential roles in determining the seed quality^5,12^. In general, seed vigour is influenced by maturity stage in harvesting time^5,7,11,13^. Seed vigour progressively increases to the point of maximum seed quality^6,14^ and then begins to decline as seeds age before harvest^15^. In present study, a significant quadratic function was observed between germination percentage (GP), vigour index (VI) and days after the completion of artificial pollination (DACAP) (Figs 1–3). Fu *et al*.^11^ showed that seed vigour was significantly correlated with seed maturation during seed development. Moreover, ΔX values of GP were higher than that of VI according to the quadratic functions (Figs 1–3). These results indicate that seed vigour is more sensitive to harvesting time than GP and confirm that seed vigor could be better used to predict the optimum seed harvesting time.

During hybrid rice seed production in China, pre-harvest sprouting is a constrain problem resulting in poor seed quality and grain yield^16^. Therefore, timely harvest is of importance for hybrid rice to achieve maximum seed viability, vigor and yield. In this study, the optimum harvesting time based on seed vigour was ranged from 17 to 23 DACAP for the production of high vigour hybrid rice seeds. Fu *et al*.^11^ reported that hybrid rice variety zhuliangyou06 could be harvested as early as 20 days after pollination with high dry weight and seed vigor during seed production. Shu *et al*.^17^ found that 23 days after pollination could be the optimal harvesting time of zhuliangyou819 based on antioxidant enzyme contents and seed vigor. The range of optimum harvesting time in this study conformed to the reported results. Moreover, GP of seedlots which harvested from the divided optimum harvesting time were higher than Chinese standard (80%)^18^. Therefore, it can be concluded that 17 to 23 DACAP can be recommended as optimum harvesting time for the production of hybrid rice seeds with high vigour.

1000-grain weight is one of yield components. In this study, the significant logistic model of 1000-grain weight was consistent with the result of Kim *et al*.^19^. Additionally, grain yield was determined by the 1000-grain weight as other components were kept at the same level in present experiment. Nevertheless, no significant difference was found on grain weight, and the 1000-grain weight of seedlots harvested (≥17 DACAP) kept stable among varieties. Moreover, Seed moisture content is a major concern during the harvesting and processing. Harvesting with a high moisture content can improve head rice yield but increases drying costs at the milling. In contrast, harvesting with a low moisture content can save in drying costs but decreases head rice yield due to fissuring^20^. In present study, the seed moisture content kept low (19–25%) when seedlots were harvested as early as from 17 to 23 DACAP. Fu *et al*.^11^ found that the optimum harvesting moisture content of zhuliangyou06 and chunyou84 was about 20% and 25%, respectively. In addition, rice with lower harvesting moisture content (<15%) leads to decreases of agronomic yield and economic benefits^21^. The seed moisture content range (19–25%) in this study was in accordance with the reported results. Consequently, the seed moisture content from 19% to 25% is suitable for hybrid rice seed harvest. These results highlights that hybrid rice seed can be earlier-harvest with the range from 17 to 23 DACAP. However, the deficiency of this study is that the results were not referred to meteorological factors, which greatly affect grain yield and yield components^22,23^. Further studies are needed regarding the influences of meteorological factors on seed quality and grain yield during hybrid rice seed production.

## Conclusions

Seed vigour is more sensitive to harvesting time than germination percentage (GP) and could be better used to predict the optimum seed harvesting time. This study highlights that hybrid rice seed can be earlier-harvest based on seed vigour, and 17 to 23 days after the completion of artificial pollination (DACAP) can be recommended as optimum harvesting time during hybrid rice seed production.

## Materials and Methods

### Rice varieties

Rice varieties used in this study viz. IIyou416 (II-32A × R416; IIY-416), Jinyou167 (Jin23A × R167; JY-167) and Yliangyou-1 (Y58S × R9311; YLY-1), collected from Longping Seed Industry co., LTD in Hunan province, China. The climate of parents’ seeds production site is moist sub-tropical monsoon.

### Field experiments

Field experiments were carried out at Changsha (28°11′ N, 113°04′ E), Hunan province, China during 2013–2014. Plots were laid out in a randomized complete block design with three replications using plot size of 200 m^2^. Different plots were isolated by plastic films to prevent biological contamination. The row ratio of restorer line and sterile line was 2:12 and rice seedlings were raised on nursery beds, and transplanted manually. Sowing and transplanting date were shown in Table 1 and the male parent sowed twice. Male and female parents were transplanted at a spacing of 16.7 cm × 30 cm and 13.3 cm × 20 cm, respectively with five and three seedlings hill^−1^. The spacing of the parents was 33.3 cm. All other cultural practices were done uniformly as per recommendation. Flowering time was recorded when 80% panicles flowered. Panicles established uniformly were hanged by using labels. The signed panicles of 500 g were harvested by hand. Samples of tested varieties were harvested at 3-interval days after the completion of artificial pollination (DACAP), until the complete mature. All the samples of tested varieties were dried to a moisture content of 13% with sunshine and stored in mesh bags at room temperature.

**Table 1:** Parameters of polynomial fit of germination percentage (GP) and vigour index (VI).

Varieties	Seed vigour	Y = Intercept + B1*X + B2*X^2^			R^2^	Tangent point		ΔX
		Intercept	B1	B2		X1	X2	
IIY-416	GP	−16.9394	10.6284	−0.2419	0.9016	2.6329	43.3026	40.6697
	VI	−2.4051	0.6772	−0.0157	0.8616	4.8790	40.2548	35.3758
JY-167	GP	37.1737	5.7126	−0.1478	0.7214	9.9395	52.0223	42.0828
	VI	−0.4915	0.9966	−0.0264	0.8167	1.4735	38.2765	36.8030
YLY-1	GP	−29.7694	12.9711	−0.3202	0.9099	3.4065	39.1029	35.6964
	VI	−3.1278	0.7940	−0.0192	0.8516	2.5036	38.1257	35.6221

IIY-416, JY-167 and YLY-1 indicate IIyou416, Jinyou167 and Yliangyou-1, respectively. R^2^ indicates regression coefficient. X1 and X2 indicate tangent points between the equation (Y = Intercept + B1*X + B2*X^2^) and the derivative equation (Y’ = B1 + 2*B2*X), respectively. ΔX indicates the value of X2 minus X1.

**Table 2:** Parameters of logistic fit of 1000-grain weight.

Varieties	Y = A2 + (A1–A2)/(1 + (X/X0)^P)				
	A1	A2	X0	P	R^2^
IIY-416	7.8932	20.0106	7.3518	4.6990	0.9294
JY-167	9.9531	21.2196	5.7251	3.2939	0.6101
YLY-1	9.6335	24.3078	7.7852	4.1875	0.7679

IIY-416, JY-167 and YLY-1 indicate IIyou416, Jinyou167and Yliangyou-1, respectively. R^2^ indicates regression coefficient.

**Table 3:** Parameters of logistic fit of seed moisture content.

Varieties	Y = A2 + (A1–A2)/(1 + (X/X0)^P)				
	A1	A2	X0	P	R^2^
IIY-416	55.1493	17.7672	7.4002	1.8956	0.9020
JY-167	46.5628	18.5335	10.7088	2.7903	0.9823
YLY-1	83.7980	−2.7474	6.2953	0.8330	0.9765

IIY-416, JY-167 and YLY-1 indicate IIyou416, Jinyou167and Yliangyou-1, respectively. R^2^ indicates regression coefficient.

**Table 4:** Sowing and transplanting date of varieties during 2013–2014.

Year	Varieties	Female/Male	Sowing date	Transplanting date
2013	IIY-416	II-32A♀	05–03	05–23
		R416♂	04–26	05–20
			05–01	05–20
	JY-167	Jin23A♀	06–10	07–06
		R167♂	05–24	06–19
			05–31	06–19
	YLY-1	Y58S♀	05-04	05–28
		R9311♂	04–20	05–15
			04–24	05–15
2014	IIY-416	II-32A♀	05-04	05–24
		R416♂	04–25	05–20
			05–01	05–20
	JY-167	Jin23A♀	06–14	07–06
		R167♂	05–25	06–20
			05–31	06–20
	YLY-1	Y58S♀	05-04	05–28
		R9311♂	04–21	05–14
			04–25	05–14

IIY-416, JY-167 and YLY-1 indicate IIyou416, Jinyou167and Yliangyou-1, respectively.

### Measurements of moisture content and 1000-grain weight

Samples of freshly harvested seeds (about 100 g per replicate) were powdered and dried at 108 °C for 48 h to determine the seeds moisture content^24^. Other samples of freshly harvested seed were dried at 80 °C for 7 days to evaluate the 1000-grain weight^25^.

### Measurements of seed germination

The germination tests were conducted within one month after the harvesting of hybrid seeds. 100 healthy seeds with three replications were surface sterilized with 0.6% (6 g/L) sodium hypochlorite solution for 15 minutes and then rinsed three times with sterile distilled water. The seeds were then placed in a plastic box (12 × 12 × 5 cm) with two sheets of filter paper, and 9 ml of distilled water was added. The seeds were germinated in a growth chamber at (30 ± 1) °C for 7 days with a 12-h light/12-h dark photoperiod. Seeds were considered to be germinated when the root length reached the seed length and shoot length reached half of the seed length^26,27^. The number of germinated seed was counted every day for 7 days. At the end of the test period (7 days), the sum of daily counts was referred to as the final germination percentage (GP) and vigour index (VI) was calculated using the method described by the equation: ${\rm{VI}}={\rm{DW}}\times \sum (Gt/t)$, where DW is the dry weight of the seedlings of germinated seeds and *Gt* is the number of the germinated seed on Day *t*^28^.

### Data analysis

Data were analyzed using the analysis of variance (ANOVA) procedure in Statistical Analysis System (SAS 8.0) software, and the differences between applications were compared using a least significant difference (LSD) test at the 0.05 probability level. Before analysis, the data of percentage were transformed according to ${\rm{y}}=\arcsin \sqrt{\frac{x}{100}}$. Figures related to curve simulation showed with integrated data of two years’ experiments.
